# Elastomer-Based Sealing O-Rings and Their Compatibility with Methanol, Ethanol, and Hydrotreated Vegetable Oil for Fueling Internal Combustion Engines

**DOI:** 10.3390/ma17020430

**Published:** 2024-01-15

**Authors:** Miroslav Müller, Rajesh Kumar Mishra, Vladimir Šleger, Martin Pexa, Jakub Čedík

**Affiliations:** Faculty of Engineering, Czech University of Life Sciences Prague, Kamycka 129, Suchdol, 165 00 Prague, Czech Republic; muller@tf.czu.cz (M.M.); sleger@tf.czu.cz (V.Š.); pexa@tf.czu.cz (M.P.); cedik.jakub@seznam.cz (J.Č.)

**Keywords:** material compatibility, methanol, ethanol, green diesel, tensile strength, O-rings

## Abstract

Green methanol, ethanol, and diesel-based hydrotreated vegetable oils are some of the renewable liquid fuels that show satisfactory performance in diesel engines. A notable advantage of these fuels is that they are renewable and do not require significant modifications in the existing engines for successful operation. Suitable fuel systems, especially their material compatibility, remain unresolved, and therefore, it is a weak link in their large-scale adaptation. Elastomer-based sealing O-rings lose their mechanical properties after a short exposure time to these fuels, adversely impacting their functionality. This research study evaluated the long-term material compatibility of different elastomer-based sealing materials by immersing the O-rings in these test fuels (hydrotreated vegetable oil, methanol, ethanol, and diesel) for different time intervals (i.e., up to 15 months). The material compatibility was assessed mainly by investigating these changes in various mechanical properties of these O-rings, namely tensile strength (Δ*T_s_*), elongation at break (Δ*E_b_*), Shore A hardness (Δ*H*), and mass (Δ*M*). The degradation of mechanical properties was studied and analyzed during the immersion interval from 0.9 to 15.2 months and compared with O-rings kept in a normal atmosphere. It was noted that individual fuels affect various mechanical properties significantly. In a short interval of 0.9 months (28 days), significant changes in the mechanical properties of the sealing O-rings were observed.

## 1. Introduction

Environmental protection is perceived as a critical global task. The impact of transportation on atmospheric pollution is significant and continuous. It has an undeniable influence on environmental deterioration, which is constantly pointed out by various regulatory bodies. A trend in developed countries is to go for electrification of transport systems, which offers great potential, especially in regions where energy is obtained from Green House Gas (GHG)-free primary renewable sources [1]. However, this trend depends on building adequate charging infrastructure and energy independence for fossil fuel-based generation of electricity. The limited capacity of the transmission grid and the lack of raw materials needed for the production of batteries are significant challenges that the world is facing in promoting the electrification of transport systems. Until all these problems associated with electrification are resolved, there is still room for environmental protection in the existing transport sector ecosystem [2]. Fuels produced from renewable resources/feedstocks are suitable for powering the transport sector, which can replace petroleum-based fuels without significant technical interventions in the current generation of internal combustion engines (ICEs). The results by Lu et al. [1] recommended that a public transport system with hybrid and electric buses can be a good trade-off between financial and environmental needs instead of using electric buses to displace all conventional buses. In addition, the operating schedule of a public transport system combined with electric and hybrid buses can be adjusted to seasonal temperature variations to minimize environmental hazards. To achieve carbon neutrality, finding a sustainable mobility solution and manufacturing a large number of vehicles that use what are now considered fossil fuels is imperative. One option is to use biofuels in existing engines/vehicles [3]. Biofuels offer excellent potential for ICEs, especially in developing/underdeveloped countries, to conserve non-renewable resources and contribute to environmental protection [4]. Electrification of transport cannot be expected in areas lacking resources for battery production [5]. Using biofuels directly in ICEs requires the resolution of several technical challenges, including their aggressiveness towards sealing elements, which are an integral part of fuel injection equipment (FIE) assemblies. Since diesel, gasoline, and other conventional fuels have been used for more than a century, their influence on elastomer-based materials is well-researched and well-known. To use biofuels in the transport sector effectively, their effects on the sealing elements of the FIE must be thoroughly investigated. Studies of conventional and biofuels in ICEs and their effect on FIE systems have been undertaken by various research groups over a long time [6].

Research into the compatibility of different biofuels and elastomers is therefore essential. The mechanical properties of elastomer-based materials change after exposure to biofuels [7,8,9,10]. The degradation and associated material compatibility of elastomer sealing rings depend on the physicochemical properties of these fuels [11]. After increasing the acrylonitrile content of elastomers, their degradation in biodiesel decreases [12]. Alves et al. [13] suggested that it does not affect the degradation of elastomers. Thomas et al. [14] found a slight variability in the degradation of elastomers with different biofuel feedstocks, such as Jatropha curcas, palm, cottonseed, and soya. The changes in hardness and tensile strength were correlated with the bulk swelling data of the tested elastomers in rapeseed biodiesel. Haseeb et al. [9] reported that a higher degree of degradation occurred in elastomer materials (tested for 750 h) with elapsed time. Alves et al. [13] reported that the short immersion duration in biofuels led to relatively small differences in the measured values of various mechanical properties. They also pointed out the potential effects of fuel in conjunction with pressure on the properties of elastomers. Zang et al. [15] reported that NBR (acrylonitrile butadiene rubber) and FKM (fluorocarbon material) are diesel-resistant elastomers that can be used in ICEs.

In contrast, SBR (styrene-butadiene rubber), rubbers based on EPDM (ethylene-propylene-diene monomer), and VMQ (vinyl-methyl silicone) are not diesel-resistant. For thermoset elastomers, e.g., NBR (Nitrite Butadiene Rubber), NBR/PVC (Nitrile Butadiene Rubber and Polyvinyl Chloride), etc., which are not resistant to diesel, biodiesel exhibited lower swelling than baseline diesel. Crouse et al. [16] reported that biodiesel causes more volumetric swelling than baseline diesel. The concentration of biodiesel was also an essential factor in the degradation process of elastomers. Frame et al. [17] reported no significant difference from baseline diesel, and all tested elastomers were found to be compatible with 20% (*v*/*v*) biodiesel blends in their study. Munoz et al. [18] reported that elastomeric materials intended for diesel were compatible with biodiesel up to a 40% (*v*/*v*) blend ratio. Chai et al. [19] reported that inelastic responses due to stress-softening (after experiencing the cyclic loading) decreased significantly as the swelling increased. This undesirable factor was critical, especially for the functioning of elastomer-sealing O-rings. Chandran et al. [20] concluded that there were differences in the results obtained from only the immersed samples vis-à-vis a modified method simulating diesel engine operation (i.e., changing temperature, length of immersion, pressure, etc.). According to Szete and Leung [3], hydrotreated vegetable oil (HVO) is an optimum solution for sustainable mobility, having a higher technology readiness level (TRL).

In the lifecycle research of conventional diesel engines, there are various convincing results in favor of renewable biofuels, either in pure form or blended with diesel. The main disadvantage of alcohol-based biofuels is their aggressiveness, especially towards rubber/elastomer-based sealing elements and hoses. For this reason, in the framework of this experimental study, the material compatibility of HVO (100% (*v*/*v*) hydrotreated vegetable oil), 100% (*v*/*v*) Methanol (M100), 100% (*v*/*v*) Ethanol (E100), and 100% (*v*/*v*) Diesel (D100) towards elastomer components, namely O-rings made of Hydrogenated Acrylonitrile Butadiene Elastomer (HNBR), Polyacrylate Elastomer (ACM), and Fluorosilicone Elastomer (FVMQ) exposed for predefined duration. It was followed by an assessment of the impact on their mechanical properties after immersion. The research study aims to assess the material compatibility of elastomeric O-rings with the above-mentioned test fuels and periodically monitor the impact of test fuels on their mechanical properties over a long duration of exposure. In the material compatibility research of the above-mentioned test fuels, the influence of liquid contaminant in the form of fuel on the degradation process were considered. Further, the process of mechanical deformation, where undesirable changes can be expected to be significant, was analyzed.

## 2. Methodology

### 2.1. Materials

Elastomer-based O-rings made from the following materials were selected for the research:

- Polyacrylate Elastomer—ACM (trade name Nipol AR^®^, Bohemia Seal, s.r.o., Prague, Czech Republic),
- Hydrogenated Acrylonitrile Butadiene Elastomer—HNBR (trade name Therban^®^, Bohemia Seal, s.r.o., Prague, Czech Republic),
- Fluorosilicone Elastomer—FVMQ (trade name Silastic^®^, Bohemia Seal, s.r.o., Prague, Czech Republic).

According to the specification of the supplier, the ACM O-rings are highly resistant against oils with a high temperature, and they are used in the automotive industry for sealing high-temperature lubrication systems such as engine and gearbox lubrication systems. The temperature range of the ACM O-rings is from −20 to 175 °C. The HNBR O-rings are highly resistant against mineral oils and their additives, and they have a temperature range from −20 to 150 °C. The FVMQ O-rings are highly resistant against fuels, oils, and solvents, especially chlorinated and aromatic hydrocarbons and alcohols; their temperature range is from −60 to 200 °C.

D100 is pure mineral diesel, E100 is pure ethanol, M100 is pure methanol, and HVO is pure hydrogenated vegetable oil, which is a mixture of paraffinic hydrocarbons. There was no unsaturated fatty acid content in the fuels.

The physicochemical properties of the test fuels are given in Table 1.

### 2.2. Methods

The material compatibility of O-rings was evaluated by immersing them in the test fuels for a duration of (i) 0.9 months (27 days), (ii) 4.2 months (126 days), and (iii) 7.8 months (234 days). The time duration was selected based on ASTM D471-16 [26], a standard test method for assessing the effects of various liquid fuels on polymer rubber properties. The determination of the other two test intervals of 9.7 months (291 days) and 15.2 months (456 days) was decided based on a continuing geometric series based on ASTM D471-16 because the applicability of these results to practical applications requires longer duration exposure for periodic replacement of the O-rings. Hence, two additional test durations were decided to conclude the material compatibility investigations. A Stabinger Viscometer (Anton Paar GmbH, Sumida, Tokyo; SVM 3000; measurement accuracy ± 1%, repeatability = 0.1%) was used to measure test fuels’ density and kinematic viscosity. The lower heating value of test fuels was measured using an isoperibol calorimeter (LECO AC600; measurement accuracy ± 0.1% RSD) following ČSN DIN 51900-1 [27] and ČSN DIN 51900-2 [28] standards. The atomic mass of molecules was used for calculating test fuels’ carbon, oxygen, and hydrogen contents. Carbon and oxygen contents for diesel and HVO were considered per DIN EN 590 [22] and DIN EN 15940 [25] standards. The cetane numbers of the test fuels were taken from other research studies. The immersion was conducted under standard atmospheric conditions, e.g., 25 ± 2 °C temperature and 65 ± 2% relative humidity.

Several properties of HVO were similar to baseline diesel, except the lower density, which can fulfill the requirements similar to baseline diesel as per DIN EN 590. Properties of alcohols (M100 and E100), such as reduced viscosity and cetane number in comparison with diesel, restrict their use as a blending component [4]. Ethanol and methanol have very low solubility in diesel as well. Therefore, using cosolvents such as iso-alcohols, dodecanol, or oleic acid, is necessary in diesel-alcohol blends.

**Figure 1:**
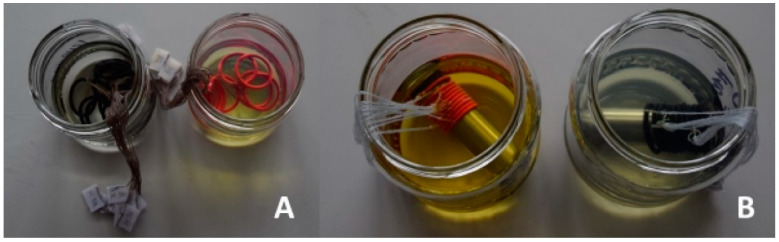
Testing of elastomer-based O-rings in fuels (**A**) loose placement, (**B**) on the mandrel.

Several studies revealed a significant effect of test fuels on elastomeric sealing elements and their mechanical properties. Undesirable changes were higher if the elastomeric materials were exposed to a degrading environment while subjected to mechanical loading. This factor was considered in the design of the experiments for this study. As part of the tests aimed at testing the effect of test fuels, the O-rings were placed for long-term monitoring as follows:

- loose placement of elastomer-based O-rings in the fuel, as shown in Figure 1A.
- elastomer-based O-rings on the mandrel in each test fuel, as shown in Figure 1B.

The objective was to simulate mechanical stress. The mandrel for the placement of elastomeric O-rings was made by chip-cutting machining from a metallurgical blank in the form of a circular drawn bar of S235JRC+C material following DIN EN 10277:10278 [29,30]. Placing the elastomeric O-ring on the mandrel created a 4.5% deformation of these sealing elements. This value corresponds to the recommended deflection for the elastomer-based O-rings.

**Figure 2:**
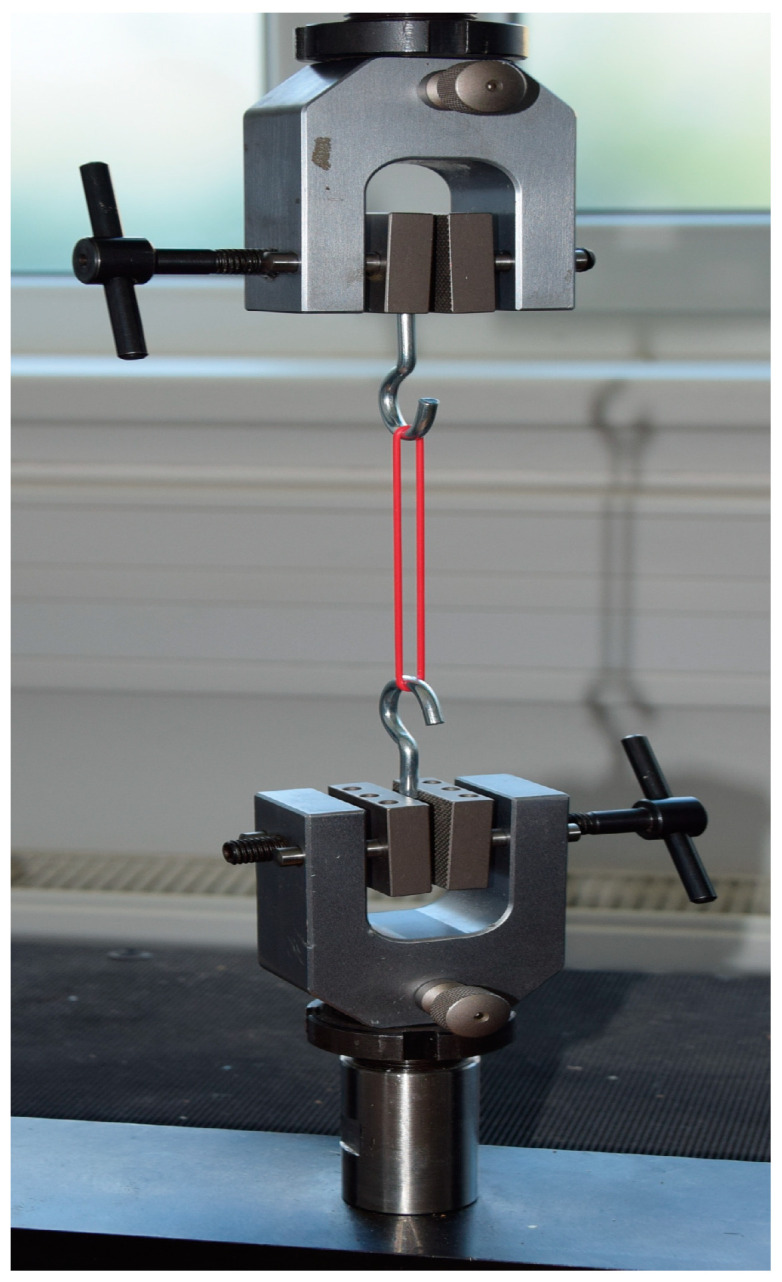
Testing of elastomer-based O-rings on the universal testing machine.

The test fuels D100, E100, and M100 are compatible with HVO, FVMQ, ACM, and HNBR. Various mechanical properties of O-rings, such as tensile strength, elongation at break, Shore A hardness, and mass, were assessed before starting the test and after the completion of the immersion duration. Besse et al. [31] and Ch’ng et al. [32] concluded that differences in material compatibility between different test fuels are best illustrated by strength characteristics and swelling levels. Once the immersion duration was over for the O-rings, various mechanical properties were measured according to ASTM D471-16 [26]. The measurements were performed after the evaporation of the test fuel. An electromechanical testing machine (LaborTech; MPTest 5.050) was used for measuring the tensile strength and elongation of O-rings. The machine complies with DIN EN ISO 7500-1 [33]: (i) 0.1 Class, (ii) 0.1 N accuracy for force gauge, and (iii) position measurement accuracy of the crossbar to 0.001 mm. This EN standard determines the tensile strength and elongation of O-rings. A modified standard ČSN ISO 37 [34] was used to determine the tensile properties of rubber. O-rings were placed in the jaws of a universal testing machine using hooks (Figure 2). Hooks made of 2.9 mm diameter circular steel wire were used to clamp the O-rings. The clamping hooks eliminated damage to the O-rings under tension during tensile testing. This prevented damage to the surface integrity of the test specimens. A strain rate of 100 mm/min was used.

Equation (1) was used for measuring the tensile strength (*T_s_*).

(1) $$T_{s}=\frac{F_{m}}{\left(\frac{2\pi h_{0}^{2}}{4}\right)}$$

where *F_m_* is the Maximum Tensile Force (N), *h*_0_ is the initial height (mm) of the O-ring, and *T_s_* in MPa. Equation (2) was used for calculating the changes in tensile strength (Δ*T_s_*).

(2) $$\Delta T_{s}=\frac{\left({T_{s}}_{1}-{T_{s}}_{0}\right)}{{T_{s}}_{0}}\cdot 100$$

where Δ*T_s_* (%) is the change in tensile strength, *T_s_*_0_ (MPa)is the initial *T_s_* of the O-ring specimen before immersion in the test fuel, and *T_s_*_1_ (MPa) is the *T_s_* of the O-ring specimen after immersion in the test fuel. Equation (3) was used to calculate the change in elongation at the break of the O-ring (Δ*E_b_*).

(3) $${\Delta E}_{b}=\frac{\left(C_{b}-C_{j}\right)}{C_{j}}\cdot 100\%$$

where Δ*E_b_* is the elongation at break (%), *C_b_* is the the final inner circumference (mm) of the O-ring, and *C_j_* is the initial inner circumference (mm) of the O-ring.

*C_j_* was calculated using the inner diameter of the O-ring, which was determined from the Hooks’s distance measured during tensile strength when the increment in measured force was started. The hook wire diameter was 2.9 mm. *C_b_* was calculated using Equation (4).

(4) $$C_{b}=(\pi \times 2.9)+(2\times 2.9)+(2\times L_{b})$$

where *L_b_* = distance of hooks when the O-ring breaks (mm) and *C_b_* = final inner circumference of the O-ring (mm).

The change in elongation at break (%), Δ*E_b_*, was calculated using Equation (5).

(5) $${\Delta E}_{b}=\frac{\left({Eb}_{1}-{Eb}_{0}\right)}{{Eb}_{0}}\times 100$$

where *Eb*_0_ is the initial elongation at break (%) of the O-ring specimen in the air before immersion in the test fuel, *Eb*_1_ is the elongation at break (%) of the same O-ring specimen in the air after immersion in the test fuel.

A weighing machine with a KERN analytical scale measurement facility was used to measure the mass of the O-ring. The mass of the O-ring was measured twice: (i) before immersion in the test fuels and (ii) after immersion in the test fuels.

Equation (6) was used for calculating the changes in mass (Δ*M*) of the O-rings.

(6) $$\Delta M=\frac{\left(M_{1}-M_{0}\right)}{M_{0}}\times 100$$

where Δ*M* is the change in mass (%), *M*_0_ is the mass (mg) of the O-ring specimen before immersion in the test fuel, and *M*_1_ is the mass (mg) of the same O-ring specimen after immersion in the test fuel.

The hardness of Shore A of elastomeric materials is also usually measured while evaluating material compatibility with fuels. Hardness measurement is quite challenging due to the thickness limitation on the material side, which is 4 mm according to the ČSN EN ISO 868 standard [35]. O-rings were layered on each other to encounter this challenge in a particular apparatus.

Equation (7) was used for the calculation of the change in hardness (Δ*H*).

(7) $$\Delta H=H_{1}-H_{0}$$

where Δ*H* is the change in hardness after immersion (ShA), *H*_0_ is the initial hardness of the O-ring specimen before immersion in the test fuel (ShA), and *H*_1_ is the hardness of the same O-ring specimen after immersion in the test fuel (ShA).

Twenty samples were used for tests under the same conditions. The average and standard deviation for each set of data were calculated. In the presented results, the average was reported. The CV% was below 4% in all cases.

## 3. Results and Discussion

D100 and biofuels (E100, HVO100, and M100) were used to evaluate the material compatibility of elastomeric O-ring seals to determine their potential impact on mechanical properties and hence their performance in the FIE system.

**Figure 3:**
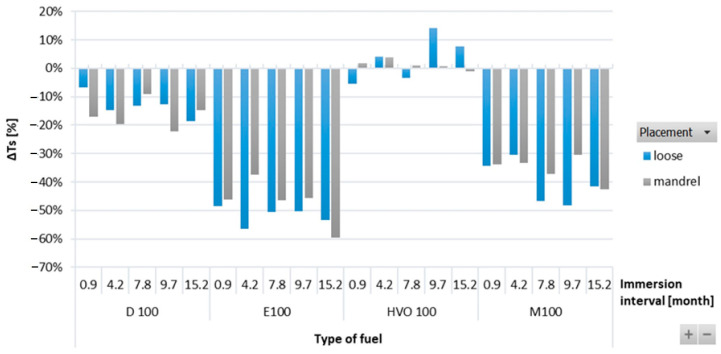
Δ*Ts* of ACM-type O-rings for all test fuels for immersion durations ranging from 0.9 to 15.2 months.

Figure 3 shows the results of Δ*T_s_* of O-rings for immersion intervals of 0.9–15.2 months. No particular trends were observed in Δ*T_s_* of the ACM type O-ring when immersed in test fuels for different durations. Significant differences in the strength of the ACM-type O-ring were recorded among different test fuels, as it highly depends on the physicochemical properties of the test fuels. The tensile strength of D100, E100, and M100 degraded and showed a negative influence of these test fuels on the O-ring strength. No such adverse impact was noticed in the case of HVO100, though. The strength of the ACM O-ring degraded by a small amount for D100 and almost 60% after immersion in E100, but it stayed at the same level for HVO. In terms of O-ring placement, no significant differences were noticed.

**Figure 4:**
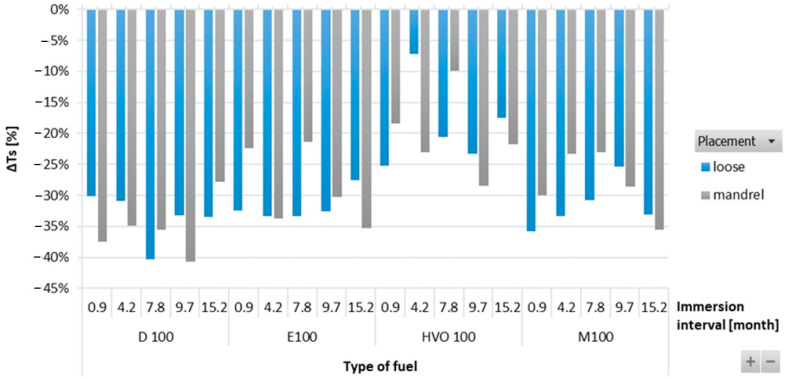
Δ*Ts* of HNBR-type O-rings for all test fuels for immersion durations ranging from 0.9 to 15.2 months.

Figure 4 shows variations in Δ*T_s_* of HNBR-type O-rings immersed in different test fuels. Δ*T_s_* was observed to be decreasing for all types of test fuels. Among all the test fuels, HVO exhibited the least impact on Δ*T_s_*. There were no significant differences observed for the position of the O-ring.

**Figure 5:**
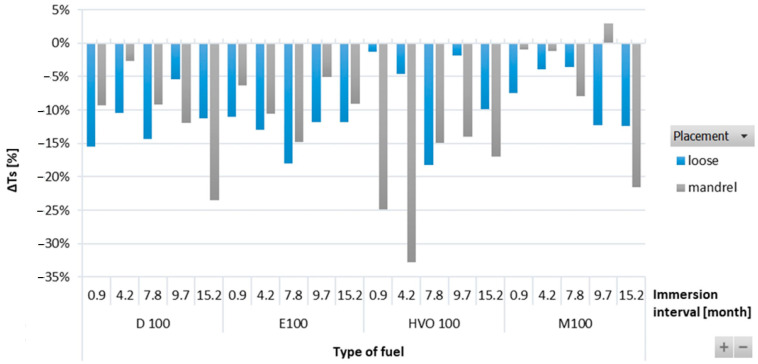
Δ*Ts* of FVMQ-type O-rings for all test fuels for immersion durations ranging from 0.9 to 15.2 months.

The variations in Δ*T_s_* of FVMQ-type O-rings for the different test fuels are shown in Figure 5. No significant trend was observed among the tensile strength and the immersion duration in the test fuels, except in M100. The tensile strength of the FVMQ-type O-ring decreased linearly with immersion duration in M100. The results showed that the tensile strength of O-rings decreased with higher exposure times for different test fuels. However, compared with other O-ring types, the lowest influence on tensile strength of the FVMQ-type O-rings was between 10 and 15%. In addition, there was also a big difference in tensile strength after immersion in HVO based on the placement of O-rings. The result showed a higher reduction in tensile strength (up to 35%) after placement on the mandrel.

In the presented results, the average of 20 samples tested under the same condition was reported. The CV% was below 4% in all cases.

**Figure 6:**
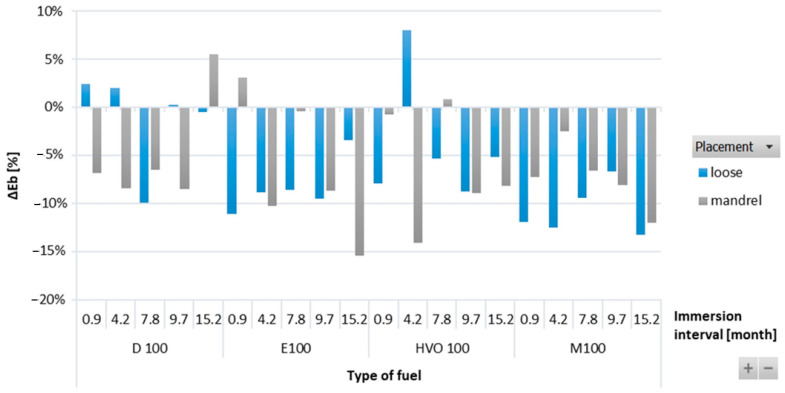
Δ*E_b_* of HNBR-type O-rings for all test fuels for immersion durations ranging from 0.9 to 15.2 months.

Figure 6 shows the negative reduction in elongation at break for HNBR-type O-rings. This decrease was directly connected to the reduction in their tensile strength. When the O-rings lose their elasticity, their strength decreases. However, a random trend of elongation at break was noticed based on the influence of immersion duration and placement of the HNBR-type O-rings.

**Figure 7:**
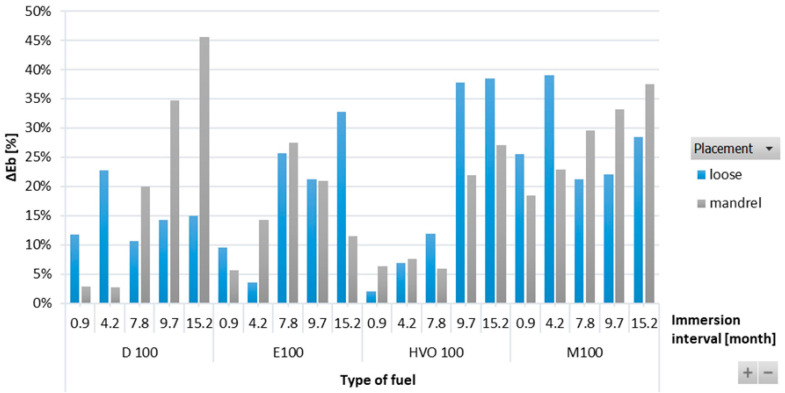
Δ*E_b_* of ACM-type O-rings for all test fuels for immersion durations ranging from 0.9 to 15.2 months.

Figure 7 shows a positive influence of immersion duration on elongation at break (Δ*E_b_*) for the ACM-type O-rings. This positive effect was also visible in the tensile strength results, where the ACM O-ring in D100 and HVO100 exhibited the lowest reduction and no significant change in tensile strength was observed. Immersion duration showed an increasing trend in elongation at break only for HVO100. However, no conclusive trend was observed for differences based on the placement of the O-ring.

**Figure 8:**
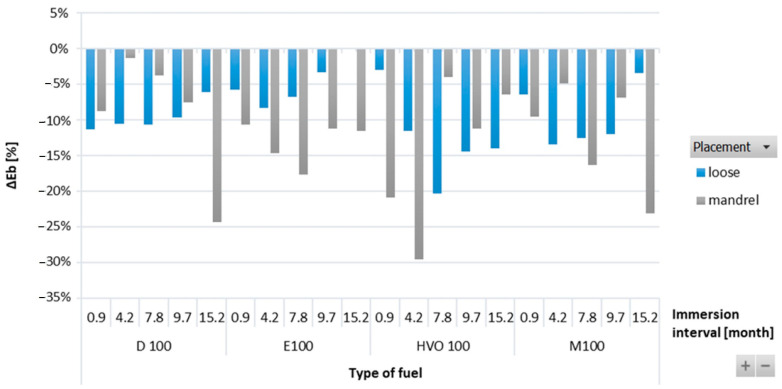
Δ*E_b_* of FVMQ-type O-rings for all test fuels for immersion durations ranging from 0.9 to 15.2 months.

Figure 8 shows a reduction in elongation at break of the FVMQ-type O-rings with immersing duration for all test fuels. The highest reduction in elongation at break was noted in the FVMQ-type O-rings after immersion in HVO100. Similarly, the highest reduction in tensile strength was noticed for immersion in HVO100. In terms of immersion duration and ring placement, no definitive trend was observed for this ring type.

**Figure 9:**
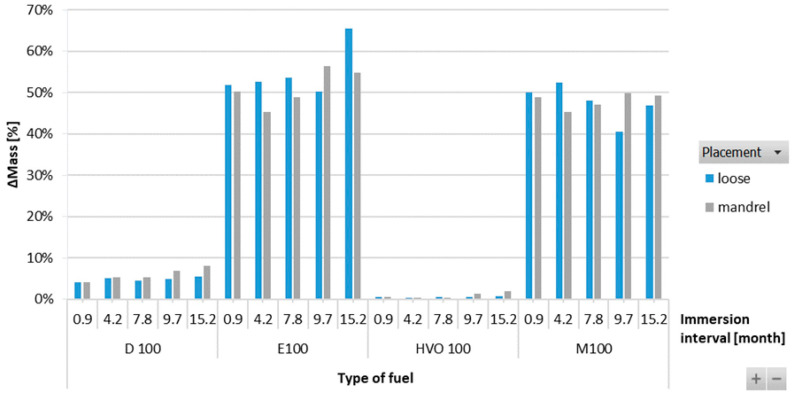
ΔMass of ACM-type O-rings for all test fuels for immersion durations ranging from 0.9 to 15.2 months.

**Figure 10:**
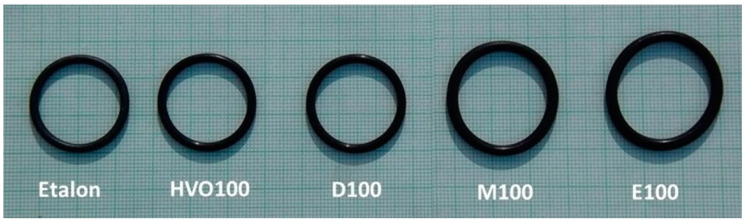
Effect of test fuels on dimensional changes of ACM-type O-rings after an immersion duration of 7.8 months.

Figure 9 shows that the ACM-type O-ring did not absorb D100 and HVO100, and their weight did not change significantly with immersion duration. This explains the superior tensile strength results of ACM-type O-rings observed in these test fuels. However, a 50% mass increment was observed for O-rings after immersion in E100 and M100. This is attributed to the reactions of the elastomers in these test fuels, leading to fuel ingress into the O-rings, leading to an increase in volume of the O-ring due to swelling, as shown in Figure 10. In terms of immersion duration and ring placement, no conclusive trend was observed.

**Figure 11:**
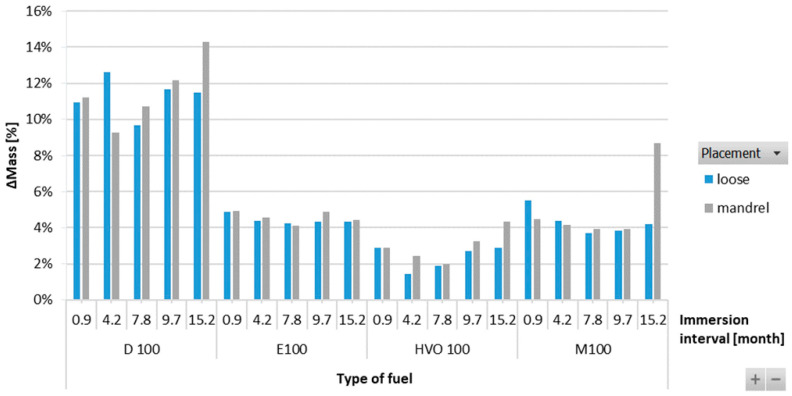
ΔMass of HNBR-type O-rings for all test fuels for immersion durations ranging from 0.9 to 15.2 months.

The results in Figure 11 showed an increase in the mass of HNBR-type O-rings for all fuel types. The highest increment, up to 12%, was observed for D100. For the biofuels, the increment in the mass of the O-rings was found to be somewhere in the range of 2 to 4%. There is no definitive trend for an increase in the mass of the O-rings with varying immersion duration and placement of the O-ring.

**Figure 12:**
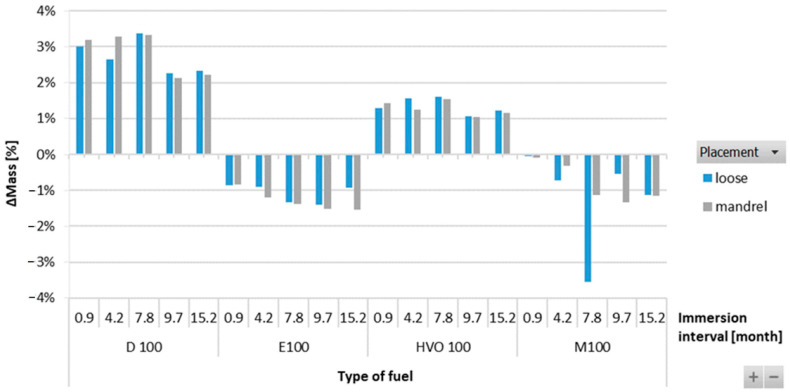
ΔMass of FVMQ-type O-rings for all test fuels for immersion durations ranging from 0.9 to 15.2 months.

**Figure 13:**
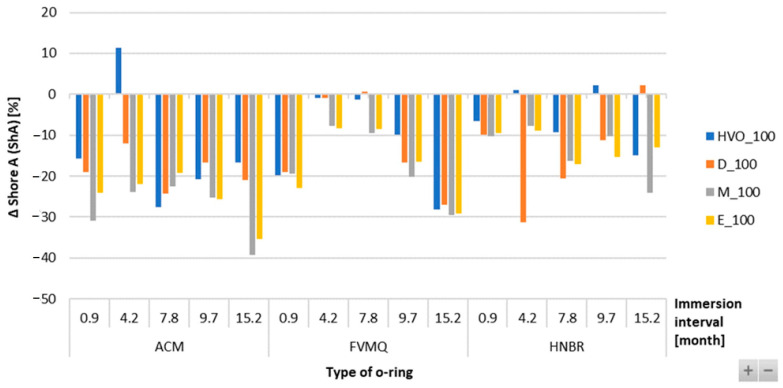
ΔShore A of all types of O-rings for all test fuels for immersion durations ranging from 0.9 to 15.2 months.

Figure 12 shows no significant difference or change in mass in the FVMQ-type O-ring with immersion duration. There is no definitive trend for an increase in the mass of the O-rings with varying durations of immersion and placement of the O-ring. Figure 13 shows a reduction in the hardness of all types of O-rings for the different test fuels with varying durations of immersion. The maximum reduction in hardness was observed for the ACM-type O-rings, which was 30%. There is no definitive trend for different types of test fuels and varying durations of immersion. An increasing trend of hardness was noticed in the case of FVMQ-type O-rings, with a longer duration of immersion.

The claim of Ch’ng et al. [32] could not be proved in this study, which indicated that swelling increased significantly when deformation was introduced. The claim of Haseeb et al. [9] related to the degradation duration of elastomeric materials also could not be proved in this study, which claimed that with increasing immersion duration, elastomeric materials had a higher degree of degradation. The conclusions of Alves et al. [13] that a short immersion duration in biofuels causes relatively small differences in the measured values were also not proven in this study.

The reasons for such observations could be due to the testing of different types of elastomers and different types of test fuels. It was proved beyond any reasonable doubt that the degradation of elastomer materials depends on the type of material used, the type of test fuel, and the duration of exposure. However, an appropriate mechanism needs to be implemented to define the degradation of elastomers after exposure to biofuels. A more detailed and comprehensive investigation is required to prepare such an appropriate mechanism.

There were no definitive trends in immersion duration in the results of this study. The elastomeric degradation process and associated material changes occurred after a very short immersion time for the practical application of O-rings in IC engines. Hence, new materials are required to be developed to overcome this issue.

The change in tensile strength Δ*T_s_* for different elastomeric O-rings and fuels varied after exposure over the time interval. From the results, it is clear that almost all the sealed fuels and elastomeric O-rings lose tensile strength *T_s_* in different time intervals. Smaller changes in tensile strength (*T_s_*) can be observed for HVO100 fuel for all three sealing elastomeric O-rings. A slight increase in tensile strength Δ*T_s_* was observed for the elastomeric O-ring type ACM placed in fuel HVO100.

The results show a clear decrease in the elongation at break ΔEb for most of the tested fuels for the elastomeric O-ring sealants FVMQ and HNBR. An increase in elongation at break Δ*E_b_* was found for almost all tested fuels for the elastomeric O-ring type ACM. In particular, these sealing O-rings showed a significant change in shape (swelling) and their elongation at break Δ*E_b_*, which is shown in Figure 10. These results correspond to a significant change in Δ*M*.

The Shore A hardness results given in the tables present the hardness loss of the sealing elastomeric O-rings after exposure to different types of fuels. Different behaviors of the tested fuels were shown, i.e., different levels of decrease in Shore A hardness. It was also shown that not only a decrease in Shore A hardness was always observed for the HVO fuel, but on the contrary, a slight increase in Shore A hardness for the elastomeric O-rings ACM and HNBR was also visible. Soot and silica fillers, which primarily serve to improve hardness and tensile properties, can be considered the cause of the decrease in the hardness of O-rings in various biofuels. These components may react with biofuels after a certain exposure time and may secondarily deteriorate the above-mentioned properties.

## 4. Conclusions

The material compatibility of O-rings made from elastomers with different test fuels was evaluated. The trend of immersion duration ranging from 0.9 to 15.2 months caused the degradation of mechanical properties in these materials. The time trend from 0.9 to 15.2 months causing the degradation process is not obvious. However, individual test fuels significantly impacted the mechanical properties of these O-rings. Significant changes in the mechanical properties of the O-rings were noticed after a short duration of immersion, i.e., 0.9 months (28 days). Swelling of the sealing elastomer-based O-rings occurred, which had a secondary effect on changes in their weight. This mass of O-rings changed significantly after their removal from the test fuel. The lowest swelling occurred for O-rings in HVO100 and D100. The test fuel’s evaporation was detected after removing O-rings from the test fuel container. The experimental results showed that the elastomer rings lost their tensile strength (Δ*T_s_*), and the elongation at break (Δ*E_b_*) decreased after immersion in the test fuels. In particular, HVO100 exhibited only minor changes in the mechanical properties of all three types of elastomer O-rings.

Among the different elastomer O-rings tested, the best performance in terms of material compatibility and dependence on the fuels was exhibited by the FVMQ (Fluorosilicone Elastomer) type O-rings. The HVO100 fuel contains hydrogenated vegetable oil, which did not significantly affect the degradation of the elastomeric O-rings. The degradation of elastomeric O-rings was more pronounced for fuels containing ethanol E100 and methanol M100. An appropriate mechanism needs to be implemented to define the degradation of elastomers after exposure to biofuels. A more detailed and comprehensive investigation is required to prepare such an appropriate mechanism. Further, the chemical degradation can be extensively studied to understand the micro-level degradation of the elastomer in the test fuels.

## Figures and Tables

**Table 1:** Basic test fuel properties [21,22,23,24,25].

Test Fuel	Kinematic Viscosity at 40 °C (mm^2^ s^−1^)	Density at 15 °C (kg m^−3^)	Calorific Value (MJ kg^−1^)	Cetane Number	Carbon Content (% wt)	Hydrogen Content (% wt)	Oxygen Content (% wt)
D100	1.878	820.67	43.2	50	87	13	0
E100	1.21	812.93	26.8	5–8	52.2	13.1	34.7
HVO100	2.905	781.87	44	>75	85	15	0
M100	0.563	797.57	19.6	<5	37.5	12.6	49.9

## Data Availability

Data are included in the article.
